# Supplementary material for: Multilevel development of cognitive abilities in an artificial neural network
Source: Proc Natl Acad Sci U S A. 2022 Sep 19;119(39):e2201304119. doi: 10.1073/pnas.2201304119 (PMC9522351; doi:10.1073/pnas.2201304119)
Supplement: Supplementary File [file pnas.2201304119.sapp.pdf]

PNAS

Supporting Information for

Multilevel Development of Cognitive Abilities in an Artificial Neural Network

Authors: Konstantin Volzhenin, Jean-Pierre Changeux, Guillaume Dumas

Corresponding author: Guillaume Dumas, Jean-Pierre Changeux

Email: guillaume.dumas@umontreal.ca, changeux@noos.fr

This file includes:

Figure S1

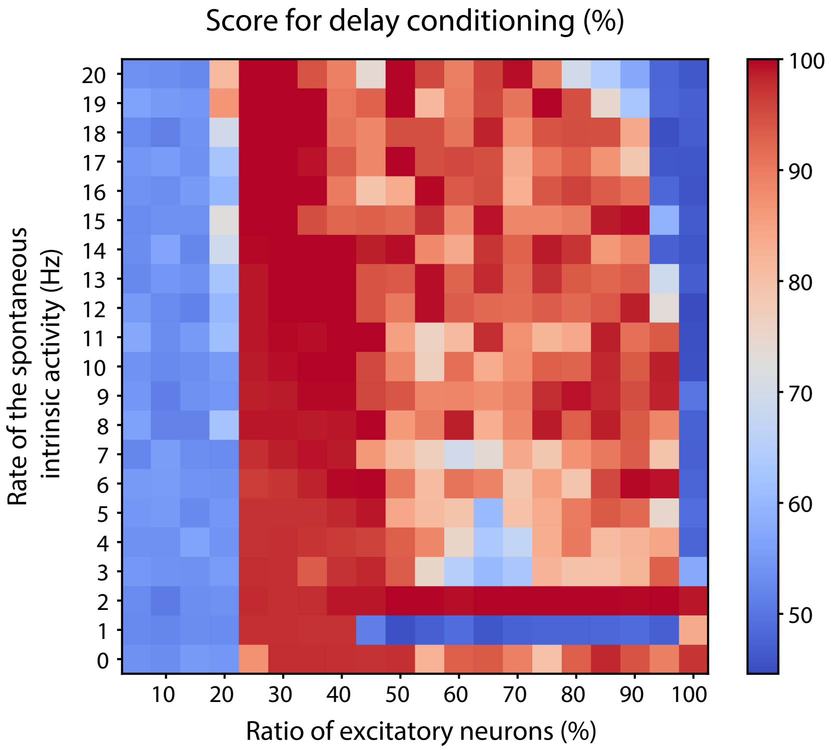

Figure S1. **Factors affecting the performance during the delay conditioning on the *cognitive level*.** The accuracy of the binary classification as a function of the rate of spontaneous intrinsic activity and the proportion of the interneurons. Notice how the optimal parameters for the trace conditioning on the Conscious Level still provide good performance for the delay conditioning.
